# Markers of kidney function, genetic variation related to cognitive function, and cognitive performance in the UK Biobank

**DOI:** 10.1186/s12882-022-02750-6

**Published:** 2022-04-27

**Authors:** Erin L. Richard, Linda K. McEvoy, Ian J. Deary, Gail Davies, Steven Y. Cao, Eyal Oren, John E. Alcaraz, Andrea Z. LaCroix, Jan Bressler, Rany M. Salem

**Affiliations:** 1 Department of Family Medicine and Public Health, University of California San Diego, 9500 Gilman Dr, La Jolla, San Diego, CA USA; 2 Department of Family Medicine and Public Health, Herbert Wertheim School of Public Health and Human Longevity Science, University of California San Diego, School of Medicine, 9500 Gilman Dr, La Jolla, CA 92093-0841 San Diego, USA; 3 Department of Radiology, University of California San Diego, 9500 Gilman Dr, La Jolla, San Diego, CA USA; 4 Lothian Birth Cohorts, Department of Psychology, University of Edinburgh, Edinburgh, UK; 5 Graduate School of Public Health, San Diego State University, 5500 Campanile Dr, San Diego, CA USA; 6 Human Genetics Center, Department of Epidemiology, School of Public Health, Human Genetics, and Environmental Sciences, The University of Texas Health Science Center at Houston, Houston, TX USA

**Keywords:** Cognitive aging, Glomerular filtration rate, Albuminuria, Polygenic score

## Abstract

**Background:**

Chronic kidney disease has been linked to worse cognition. However, this association may be dependent on the marker of kidney function used, and studies assessing modification by genetics are lacking. This study examined associations between multiple measures of kidney function and assessed effect modification by a polygenic score for general cognitive function.

**Methods:**

In this cross-sectional study of up to 341,208 European ancestry participants from the UK Biobank study, we examined associations between albuminuria and estimated glomerular filtration rate based on creatinine (eGFRcre) or cystatin C (eGFRcys) with cognitive performance on tests of verbal-numeric reasoning, reaction time and visual memory. Adjustment for confounding factors was performed using multivariate regression and propensity-score matching. Interaction between kidney function markers and a polygenic risk score for general cognitive function was also assessed.

**Results:**

Albuminuria was associated with worse performance on tasks of verbal-numeric reasoning (β(points) = -0.09, *p* < 0.001), reaction time (β(milliseconds) = 7.06, *p* < 0.001) and visual memory (β(log errors) = 0.013, *p* = 0.01). A polygenic score for cognitive function modified the association between albuminuria and verbal-numeric reasoning with significantly lower scores in those with albuminuria and a lower polygenic score (*p* = 0.009). Compared to participants with eGFRcre ≥ 60 ml/min, those with eGFRcre < 60 ml/min had lower verbal-numeric reasoning scores and slower mean reaction times (verbal numeric reasoning β = -0.11, *p* < 0.001 and reaction time β = 6.08, *p* < 0.001 for eGFRcre < 60 vs eGFRcre ≥ 60). Associations were stronger using cystatin C-based eGFR than creatinine-based eGFR (verbal numeric reasoning β = -0.21, *p* < 0.001 and reaction time β = 11.21, *p* < 0.001 for eGFRcys < 60 vs eGFRcys ≥ 60).

**Conclusions:**

Increased urine albumin is associated with worse cognition, but this may depend on genetic risk. Cystatin C-based eGFR may better predict cognitive performance than creatinine-based estimates.

**Supplementary Information:**

The online version contains supplementary material available at 10.1186/s12882-022-02750-6.

## Background

According to the United Nations, older individuals (ages 65 and above) comprise the fastest growing segment of the global population [1]. Older age is a significant risk factor for cognitive decline and the global burden of dementia and cognitive impairment is expected to rise exponentially as a result [2]. While cognitive decline is a natural consequence of aging, there is considerable variability in cognitive function decline with age [3]. Along with increasing age, cognition is also influenced by genetics [4, 5], lifestyle factors [6, 7] and chronic health conditions such as diabetes, hypertension, and kidney disease [8, 9].

Chronic kidney disease (CKD) is also increasing in prevalence. The global all-age prevalence of CKD has increased by almost 30% over the past few decades [10]. Impaired kidney function is typically detected by decreased estimated glomerular filtration rate (eGFR) or by albuminuria (the presence of albumin protein in the urine indicative of glomerular damage). There is a growing body of evidence supporting an association between albuminuria and decreased cognitive ability [11–13], but the relationship between eGFR and cognition has been mixed [14–16]. Of the latter studies, the majority use eGFR based on serum creatinine concentrations (eGFRcre) which is highly dependent on sex, age, and muscle mass [17]. Cystatin C based eGFR (eGFRcys) has received considerably less attention in epidemiological studies, likely due to the increased measurement cost relative to that of creatinine. However, being a ubiquitous small protein, cystatin C is less influenced by muscle mass and has been shown to be a better predictor of end-stage renal disease (ESRD) and cardiovascular events compared to creatinine [18, 19]. Likewise, some studies suggest eGFRcys may be a relevant prognostic factor for worse cognition [20] and incident dementia [21], but studies that consider all three measures of kidney function are still lacking. Moreover, the extent to which these associations are modified by genetic predictors of cognitive function has not been sufficiently studied.

Genetic factors play a substantial role in determining individual differences in global cognitive ability with twin-based heritability estimates of over 50% [22]. The potential for genetics to modify the effects of environmental exposures on cognitive function is also of growing interest [23]. For example, studies have shown that associations between cognitive decline and factors such as type 2 diabetes and social engagement were dependent on polygenic risk for Alzheimer’s disease [24, 25]. Likewise, between-individual cognitive differences related to kidney disease may be affected by genetic determinants of cognitive function. That is, genetic factors may confer a degree of resistance or susceptibility to the effects of kidney disease on the brain. We hypothesized that genetics and impaired kidney function may jointly influence cognitive performance. Here we leveraged UK Biobank data to investigate the associations between eGFRcre, eGFRcys and albuminuria with cognitive performance and evaluated potential modification by a polygenic score for global cognitive function.

## Methods

### Study population

A cross-sectional study was performed using data from the UK Biobank (UKBB), a large prospective cohort that enrolled 502,617 participants aged 40–73 years from across the United Kingdom between 2006 and 2010. UKBB was designed and conducted with data sharing in mind, providing researchers access to genotypic and phenotypic data [26]. Details of enrollment procedures have been previously described [27]. Participants completed a detailed, computerized questionnaire at baseline that included a wide range of information pertaining to lifestyle and health characteristics. A series of cognitive function tests was administered via touchscreen at this time. Biospecimen samples were collected for the full cohort and stored for biochemical tests and genotyping. In addition, study data was linked to participants’ national health records for longitudinal follow-up.

### Genotyping

The UKBB study was genotyped on the Affymetrix (now part of ThermoFisher Scientific) UK BiLEVE Axiom array (*n* = 49,950 participants) or the similar UKBB Axiom array (*n* = 438,427). To facilitate use of the UKBB resource by the research community, genotyping, quality control (QC) and genotype imputation were performed centrally by the primary UKBB investigators as described by Bycroft et al. [28]. Genotype imputation is a statistical technique that leverages directly genotyped variants and a reference panel to infer ungenotyped variants. Prior to imputation, genetic data from the two arrays were combined and a QC procedure performed. Poor quality markers were identified using statistical tests for inconsistencies of genotype calling across experimental factors, including batch effects, plate effects, departures from Hardy–Weinberg equilibrium, sex effects, array effects, and discordance across control replicates. Post quality control, genetic data is available for 488,377 subjects on 805,426 genetic markers and 92,693,895 imputed variants. We carried out the following additional quality control and filtering steps. Individuals with the following characteristics were excluded: extreme heterozygosity or missingness (*n* = 968), individuals with sex chromosome aneuploidy (*n* = 651), individuals whose reported sex did not match genetically inferred sex (*n* = 186), and individuals with high levels of cryptic relatedness (*n* = 73). Principal components were then calculated for the remaining 486,387 participants using 1000 Genomes as the reference population [29]. We used the “aberrant” clustering package in R [30] with a lambda parameter of 8.2 to determine the European ancestry cluster. Subjects with self-report of non-British or non-European ancestry included in the European ancestry cluster were excluded, resulting in, 454,488 participants with European ancestry. To avoid inflation in test statistics due to inclusion of related individuals, we used a custom script that implements a greedy algorithm to determine the unrelated subset. Relatedness was first determined by UKBB using identity by state (IBS). The algorithm sequentially breaks related pairs to retain only unrelated individuals while preferentially maximizing the number of individuals with a user defined characteristic. In this study we chose to maximize those with available verbal-numeric reasoning scores. We excluded those with approximately second degree or closer relatedness (pi-hat = 0.0884, *n* = 69,378 removed). After additionally excluding those who had withdrawn consent at the time of this study, pregnant women (*n* = 119), individuals with probable type 1 diabetes (*n* = 1,670) and participants missing data on kidney function exposures or covariates included in multivariate models there remained 118,146, 340,887 and 341,208 participants for analyses with verbal-numerical reasoning, reaction time, and visual memory scores, respectively.

### Kidney function markers

Blood and spot urine samples were collected and analyzed at the initial assessment (2006–2010) at a centralized laboratory. Sampling, handling, and quality control of biochemical measures have been described in detail previously [31]. Briefly, serum creatinine (Field ID 30700), urine creatinine (Field ID 30510) and urine albumin (Field ID 30500) were measured on a Beckman Coulter AU5800 instrument. An enzymatic, IDMS-traceable method was used to measure serum and urine creatinine. Urine albumin was quantified using an immune-turbidimetric method (Randox laboratories) with a lower limit of detection of 6.7 mg/L. Individuals with urine albumin concentrations below this limit were considered normoalbuminuric. The urine albumin to creatinine ratio (ACR) was calculated as urine albumin in milligrams divided by urine creatinine in millimole. Albuminuria was defined as an ACR ≥ 2.5 mg/mmol for men and ACR ≥ 3.5 mg/mmol for women. Serum cystatin C (Field ID 30720) was measured on a Siemens ADVIA 1800 instrument using an Immuno-turbidimetric assay. Estimated GFR was calculated using creatinine (eGFRcre) or cystatin C (eGFRcys) by the CKD-EPI Eq. [32, 33]. Individuals with ESRD (*n* = 405) were not excluded from this analysis.

### Cognitive function

Cognitive function was assessed using a battery of self-administered, computerized tests that were specifically designed for the UKBB [34, 35]. The verbal-numeric memory, reaction time and visual memory tests were used in this analysis and are described briefly below:

#### Verbal-numeric reasoning

This test was added part-way through the initial assessment period and therefore was administered to a subset (33%) of those who participated in the baseline visit (Field ID 20016). Characteristics of individuals with and without verbal-numeric reasoning scores is available in Table S1. This test included 13 logic/reasoning-type questions. The score was the number of questions answered correctly within a two-minute time limit. The Cronbach alpha coefficient for this test, which is a measure of internal consistency, has been described as moderate (Cronbach's alpha = 0.62) [36].

#### Reaction time

Similar to the card game “Snap”, participants were shown a series of card pairs with symbols on them and were instructed to press a large button as quickly as possible when the cards matched (Field ID 20023). The score was the mean time, in milliseconds (ms), to press the button across all test trials (*N* = 4) with a matching pair.

#### Visual memory

The “pairs-matching” test was used to assess episodic visual memory in the UKBB (Field ID 100030). Participants were briefly shown the positions of six card pairs and were then asked to match them from memory in as few attempts as possible. The score on this test was the number of errors made. Pairs matching scores were log(+ 1) transformed for analyses.

### Covariates

Coronary heart disease (CHD), heart failure, and stroke were determined by self-report from a nurse-administered verbal interview or by the presence of relevant inpatient diagnostic or procedural codes from the patient’s electronic health record prior to the time of enrollment (Table S2). Menopausal status (Field ID 2724), cancer history (Field ID 20001), hyper- and hypothyroidism (Field IDs 130701 and 130697) were self-reported by verbal interview. Type 2 diabetes mellitus (T2DM) was based on a combination of self-report, diabetic medication use, and lab values. Type 1 and type 2 diabetes were first differentiated according to an algorithm developed by Eastwood et al. [37]. Individuals identified by this algorithm and those with a random plasma glucose of 11.1 mmol/l or higher or an HbA1c of 48 mmol/mol or higher were considered as having T2DM. ESRD (Field ID 42027) was determined by a predefined algorithm [38]. Participants self-reported use of any hormone replacement therapy, cholesterol lowering drugs or antihypertensive medications (Field IDs 6153 and 6177, yes/no). Smoking (Field ID 20116; never, previous, current) and alcohol consumption (Field ID 20117; never, previous, current) were also determined by self-report. Physical activity was based on metabolic equivalents (MET)-minutes per week (Field ID 22040) calculated based on walking, or moderate or vigorous physical activity. Missing values of MET-minutes per week were imputed using the age and sex specific mean value. Body mass index (BMI; Field ID 21001) was measured by trained research staff and calculated as: weight (kg)/(height (m)^2^). Low density lipoprotein cholesterol (LDL-C; Field ID 30780) was measured using a direct homogeneous Beckman assay. Triglycerides (Field ID 30870) were measured by GPO-POD using the AU5800 by Beckman Coulter. Hypertension was defined as systolic blood pressure (Field IDs 93 and 4080) ≥ 140 mmHg or diastolic blood pressure (Field IDs 94 and 4079) ≥ 90 mmHg, self-report of a past hypertension diagnosis (Field ID 6150) or use of antihypertensive medications. Townsend socioeconomic deprivation scores (Field ID 189) were based on postcode of residence with higher scores equating to higher levels of deprivation [39]. We used years of education as a continuous variable by mapping each of the educational qualifications (Field ID 6138) reported by UKBB participants to categories defined in the 1997 International Standard Classification of Education (ISCED) and imputing the number of years of schooling as described by Okbay et al. [40]. Whole body fat-free mass (Field ID 23101) was measured using bioelectrical impedance analysis with the Tanita BC418MA body composition analyzer (Tanita, Tokyo, Japan).

### Polygenic score calculation

We derived a polygenic score for cognitive function (PGScog) based on summary statistics from a meta-analysis of genome-wide association studies for general cognitive function using data from the Cohorts for Heart and Aging Research in Genomic Epidemiology (CHARGE), and the Cognitive Genomics Consortium (COGENT) consortia. Descriptive characteristics of the cohorts included in this meta-analysis are available in Supplementary Data 1. All individuals were of European ancestry. Phenotyping and genotyping methods including cohort-specific quality control procedures, imputation methods, and covariates have been described previously [4, 41]. To harmonize the cognitive function phenotype across cohorts, CHARGE and COGENT applied principal component analysis to scores from multiple cognitive tasks to extract a single measure of general cognitive function. Only cohorts with a minimum of three cognitive tests were included. Meta-analysis was performed using the METAL package [42] with a sample-size weighted model. It should be noted that UK Biobank participants were excluded from this meta-analysis to minimize bias due to sample overlap. Lead single nucleotide polymorphisms (SNPs) (*n* = 108) associated with cognitive ability at the *p* = 1 × 10e-^5^ level in the meta-analysis were used to construct the PGScog. Imputation quality scores (MACH r2 calculated by PLINK 2.0 [43]) exceeded 0.80 for all SNPs. Summary statistics and imputation quality scores for each SNP are available as supplementary data 2. For each participant, PGScog was calculated as a weighted sum of the dosage of the effect allele multiplied by the parameter estimate associated with each individual SNP using a custom script in R. Allele dosage was used to incorporate genotype uncertainty. PGScog was standardized to a Z-score where higher values indicate higher genetically-determined general cognitive ability.

### Statistical analysis

We used multivariate linear regression to assess associations between measures of kidney function (albuminuria vs normoalbuminuria, eGFRcre < 60 vs eGFRcre ≥ 60, and eGFRcys < 60 vs eGFRcys ≥ 60, where eGFR ≥ 60 reflects the normal range of kidney filtration) as predictive variables and cognitive test scores as response variables. Potential effect modification by the polygenic score for general cognitive function, sex and age were assessed by adding two-way interaction terms with each of these variables and the kidney function exposure to the model. A three-way kidney function exposure by polygenic score by sex interaction was also evaluated. Formal interaction tests were conducted using the polygenic score as a continuous Z-score. However, to illustrate interactions PGScog was divided into low (lowest quintile), medium (quintiles 2–4) and high (highest quintile) groups as done previously [44]. Interactions with a likelihood ratio test *p*-value < 0.05 were considered significant. Adjustment variables were chosen based on prior studies relating them to both the kidney function markers and cognitive function [45–47]. All models were adjusted for age, sex, education, physical activity, hypertension, T2DM status, BMI, antihypertensive and cholesterol lowering medications, the Townsend Deprivation Index, smoking, alcohol drinking, and country of birth (UK or non-UK). To examine the effects of comorbid cardiovascular disease on these associations, we repeated these analyses with additional adjustment for coronary artery disease, stroke history and heart failure. In models testing for interaction with PGScog, we additionally adjusted for the first 10 ancestry principal components to account for subtle population structure. Following the suggestion of Rothman et al. [48], we show uncorrected p-values and *p* < 0.05 was considered statistically significant. However, *p*-values below the Bonferroni adjusted significance threshold of 0.0056 are indicated in the supplementary tables.

As an additional approach to covariate adjustment, we carried out analyses after matching on propensity scores for each kidney function exposure. Logistic regression was used to estimate the propensity for each kidney function exposure based on age, sex, education, physical activity, hypertension, T2DM status, BMI, antihypertensive and cholesterol lowering medications, the Townsend Deprivation Index, smoking, alcohol drinking, and country of birth (UK or non-UK). We matched exposed to unexposed individuals at a 1:2 ratio using a greedy nearest neighbor method with the MATCHIT package in R [49]. The overall quality of the matched sample was assessed by comparing the standardized mean differences of all covariates and by visual inspection of propensity score distributions between unmatched and matched samples (Figures S1-3).

### Sensitivity analyses

We repeated multivariate analyses under the following conditions: 1. restricted to post-menopausal women adjusting for use of hormone replacement therapy (*n* = 60,869, 175,677, 175,847 for verbal-numeric reasoning, reaction time, and visual memory analyses, respectively), 2. excluding individuals with a history of stroke (*n* = 116,382, 335,407, and 335,719), 3. excluding individuals with T2DM (*n* = 112,361, 324,310, and 324,612), 4. adjusted for other measures of kidney function (i.e. associations between eGFRcys and cognitive performance were adjusted for albuminuria) 5. adjusted for triglycerides and LDL-C, 6. models with eGFRcys were additionally adjusted for self-reported history of cancer, hyperthyroidism or hypothyroidism as these conditions can influence cystatin C concentrations, 7. adjusting for whole body fat-free mass as a surrogate for total muscle mass, and 8. adjusting for potential nonlinear effects of age by adding age^2^ to the model. All analyses were carried out using R in Version 3.6.1.

## Results

Summary characteristics of participants according to sex are displayed in Table 1. The population was 54% female, and the mean age was 56.7 years (median = 58 years). According to the criteria described in the methods, there were 17,006 (5%) individuals with albuminuria, 7,605 (2.2%) with eGFRcre < 60 ml/min, and 14,986 (4.4%) with eGFRcys < 60 ml/min. A Venn diagram illustrating the overlap between the three kidney function markers shows few individuals fit all three criteria (*n* = 1175; supplemental figure S4). On average, participants had a mean verbal-numeric reasoning score of 6.17 (standard deviation (SD) = 2.10), a mean reaction time of 555 ms (SD = 113 ms), and a median of 4.11 (IQR = 3.26) incorrect answers on the visual memory task. Participant characteristics by each kidney function exposure are shown in supplementary Tables S3-S5.

**Table 1:** Characteristics of study population overall and according to sex

	**All Participants**	**Female**	**Male**
	*n* = 341,208	*n* = 183,822	*n* = 157,386
Age (years)	56.69 (8.01)	56.50 (7.91)	56.91 (8.11)
Smoking status			
Current	34,882 (10.2%)	16,086 (8.8%)	18,796 (11.9%)
Never	184,846 (54.2%)	108,045 (58.8%)	76,801 (48.8%)
Past	121,480 (35.6%)	59,691 (32.5%)	61,789 (39.3%)
Some university education	193,791 (56.8%)	100,608 (54.7%)	93,183 (59.2%)
Alcohol drinking status			
Current	319,390 (93.6%)	169,577 (92.3%)	149,813 (95.2%)
Never	10,307 (3.0%)	7,725 (4.2%)	2,582 (1.6%)
Past	11,511 (3.4%)	6,520 (3.5%)	4,991 (3.2%)
Body mass index (kg/m^2^)	27.34 (4.73)	26.94 (5.11)	27.80 (4.20)
LDL-c (mmol/L)	3.57 (0.87)	3.64 (0.87)	3.49 (0.86)
Triglycerides (mmol/L)	1.75 (1.02)	1.55 (0.85)	1.98 (1.14)
Hypertension	188,082 (55.1%)	88,941 (48.4%)	99,141 (63.0%)
Type II diabetes	16,596 (4.9%)	6004 (3.3%)	10,592 (6.7%)
Coronary artery disease	12,044 (3.5%)	2569 (1.4%)	9,475 (6.0%)
History of stroke	5,489 (1.6%)	2264 (1.2%)	3,225 (2.0%)
Heart failure	947 (0.3%)	231 (0.1%)	716 (0.5%)
Cholesterol-lowering medication	57,130 (16.7%)	22,065 (12.0%)	35,065 (22.3%)
Antihypertensive medication	68,615 (20.1%)	30,859 (16.8%)	37,756 (24.0%)
Hormone replacement therapy	NA	13,325 (7.5%)	NA
Albuminuria	17,006 (5.0%)	6,886 (3.7%)	10,120 (6.4%)
eGFRcre < 60 ml/min	7,605 (2.2%)	4,071 (2.2%)	3,534 (2.2%)
eGFRcys < 60 ml/min	14,986 (4.4%)	7,882 (4.3%)	7,104 (4.5%)
Verbal-numeric reasoning score	6.17 (2.10)	6.07 (2.03)	6.32 (2.18)
Reaction time (ms)	555.14 (113.15)	563.14 (113.51)	545.80 (112.01)
Visual memory score (errors)	4.11 (3.26)	4.11 (3.18)	4.10 (3.35)

*Abbreviations:* *eGFRcre *creatinine-based estimated glomerular filtration rate, *eGFRcys *cystatin C-based estimated glomerular filtration rate, *LDL-C *LDL-cholesterol, *ms* milliseconds

Values are shown as n (%) for categorical variables and mean (SD) for continuous variables

Albuminuria was defined as a urine albumin to creatinine ratio (ACR) ≥ 2.5 mg/mmol for men and ACR ≥ 3.5 mg/mmol for women

All characteristics are significantly different by sex except eGFRcre < 60 ml/min (*p*-value = 0.55)

### Albuminuria and cognitive function

**Fig. 1:**
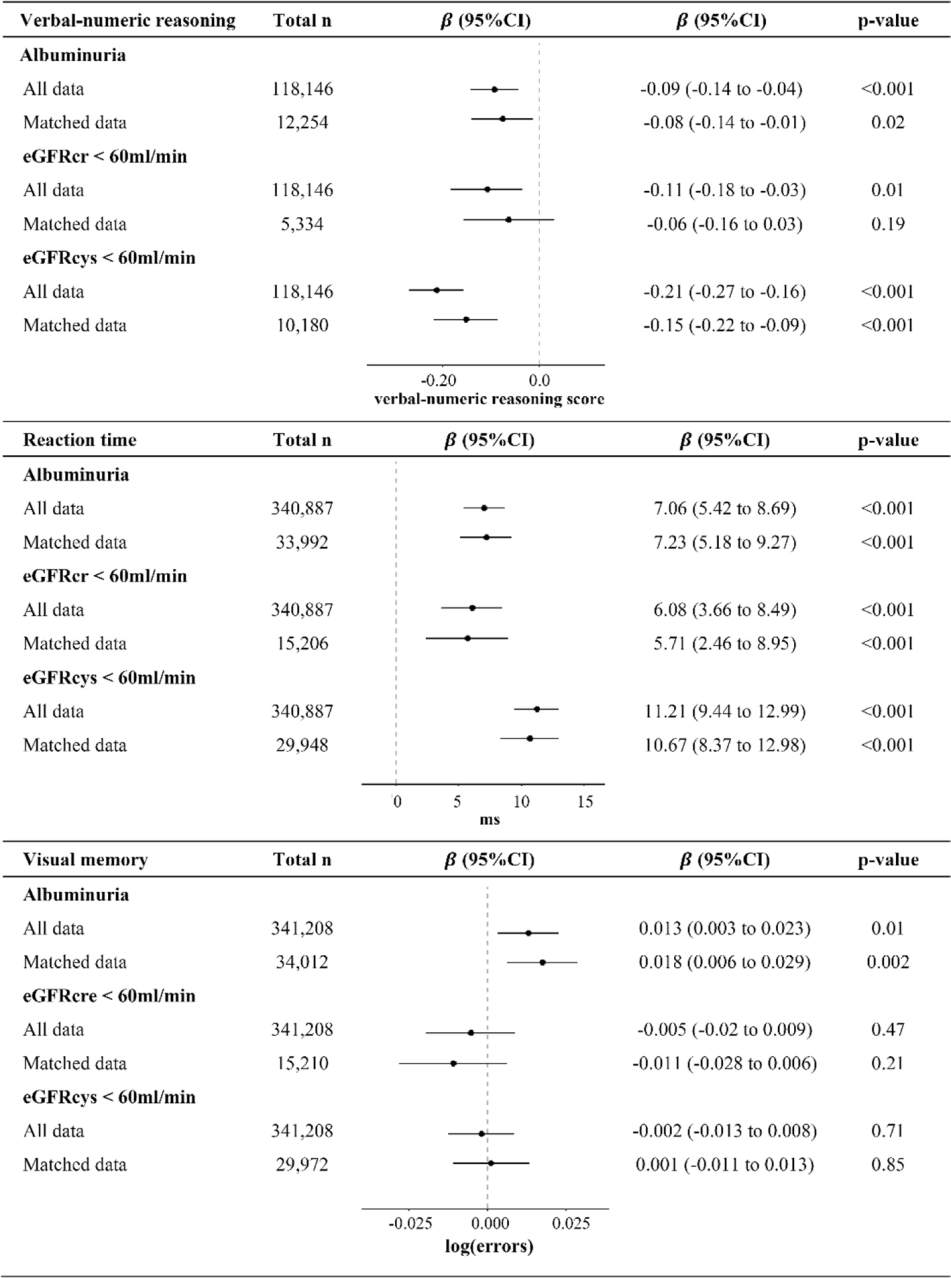
Adjusted beta estimates and 95% confidence intervals for association between kidney function and cognitive performance. Models using all data were adjusted for age, sex, education, Townsend deprivation index, country of birth, physical activity, hypertension, diabetes, alcohol use, smoking, body mass index, lipid lowering and antihypertensive drugs. Matched data based on 1:2 propensity score matching was based on the same covariate set as models using all data. Albuminuria was defined as ACR ≥ 2.5 mg/mmol for men and ACR ≥ 3.5 mg/mmol for women. Abbreviations: eGFRcre, creatinine-based estimated glomerular filtration rate; eGFRcys, cystatin C-based estimated glomerular filtration rate

Unstandardized beta estimates and 95% confidence intervals (95% CI) for the association between kidney function biomarkers and cognitive test performance among all available subjects and propensity score matched subsets are reported in Fig. 1. In multivariate analyses using all available data, albuminuria was significantly associated with lower verbal reasoning scores (β(points) = -0.09, 95% CI: -0.14 to -0.04, *p* < 0.001), slower reaction time (β(ms) = 7.06, 95% CI: 5.42 to 8.69, *p* < 0.001) and more visual memory errors (β(log errors) = 0.013, 95% CI: 0.003 to 0.023, *p* = 0.01). Regression analysis in matched subsets revealed similar results, though the magnitude of the association between albuminuria and visual memory was slightly larger (β = 0.018, 95% CI: 0.006 to 0.029, *p* = 0.002). Results of multivariate analysis in all available subjects overall and stratified by sex are shown in Table S6. We found no significant interactions with sex or age. Beta estimates for verbal-numeric reasoning and visual memory were essentially unchanged after adjustment for cardiovascular disease factors (Table S7). However, the association between albuminuria and reaction time was slightly attenuated (β = 5.54, 95% CI: 3.03 to 8.05, *p* < 0.001).

### eGFRcre and cognitive function

In the multivariate analyses using all available subjects, we found significant associations between eGFRcre category and both verbal-numeric reasoning and reaction time scores (β(points) = -0.11, 95% CI: -0.18 to -0.03, *p* < 0.001 and β(ms) = 6.08, 95% CI: 3.66 to 8.49, *p* < 0.001, respectively; Fig. 1, Table S6). However, there was no significant difference in verbal-numeric reasoning score according to eGFRcre category in matched analysis. We detected a significant sex interaction whereby eGFRcre was associated with verbal-numeric reasoning in men (β(95%CI) = -0.18(-0.29 to -0.07), *p* = 0.002) but not in women (β(95%CI) = -0.05 (-0.15 to 0.05), *p* = 0.32, p for interaction = 0.01). Associations were slightly attenuated but remained significant after adjustment for cardiovascular disease factors (Table S7). There was no significant association between eGFRcre < 60 and visual memory score. Associations were not modified by age.

### eGFRcys and cognitive function

Participants with eGFRcys < 60 performed significantly worse on verbal-numeric reasoning and reaction time tests in analyses including all available subjects (β(points)(95%CI) = -0.21(-0.27 to -0.16), *p* < 0.001 and β(ms) = 11.21(9.44 to 12.99), *p *< 0.001, respectively, Fig. 1; Table S6). Matched analyses revealed similar results. There was a significant interaction between eGFRcys category and age for reaction time (p for interaction = 0.004). To illustrate this interaction, participants were categorized as younger than the median age of 58 years or as 58 years or older. As shown in Figure S5, reaction time was significantly slower with eGFRcys < 60 in both older and younger age groups, however the association was strongest in younger individuals (β(ms)(95%CI) = 8.01(12.7 to 3.35), i.e. for those < 58 years vs ≥ 58 years).

### Kidney function by PGScog interaction

**Fig. 2:**
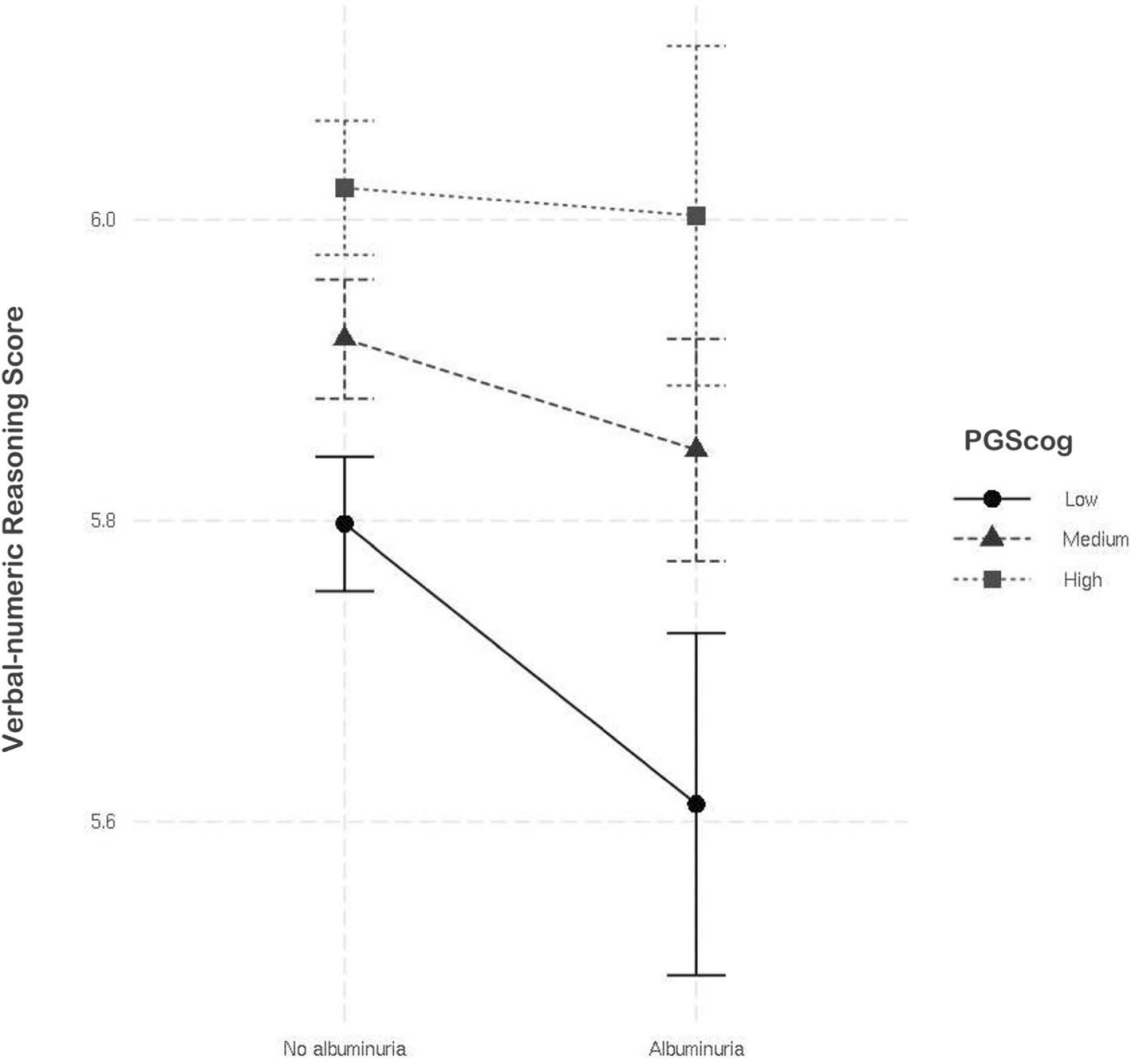
Predicted mean verbal-numeric reasoning score according to albuminuria status and cognitive function polygenic score category. For illustration, PGScog was divided into low (lowest quintile), medium (quintiles 2–4) and high (highest quintile) groups. Bars indicate 95% confidence interval. Abbreviations: PGScog, polygenic score for general cognitive function

PGScog was significantly associated with verbal-numeric reasoning (β(points) (95%CI) for highest vs lowest quintile of PGScog = 0.30(0.26 to 0.35), *p*-value < 0.0001; variance explained = 0.26%), reaction time (β(ms)(95%CI) = -2.18(-3.23 to -1.08), *p*-value < 0.001; variance explained = 0.01%) and visual memory (β(log errors)(95%CI) = -0.020 (-0.027 to -0.014), *p*-value < 0.001; variance explained = 0.01%) in models adjusted for age, sex country of birth and principal components. Significant interaction effects were observed between albuminuria and the continuous PGScog score for verbal-numeric reasoning (*p*-value = 0.009 in fully adjusted models). We did not detect any significant interactions between PGScog and eGFRcre < 60 or eGFRcys < 60 and performance on any cognitive test. Table S8 shows the results of regression analyses for model 1 including: the main effects of albuminuria and PGScog adjusted for age, sex, country of birth, and principal components; model 2: model 1 + the albuminuria by PGScog interaction term; model 3: model 2 + education and Townsend deprivation score; model 4: model 3 + physical activity, hypertension, diabetes status, alcohol use, smoking status, body mass index, lipid lowering drugs, and antihypertensive drugs; and model 5: model 4 + age^2^. Associations between albuminuria and decreased verbal-numeric reasoning scores were stronger among individuals with a lower polygenic risk score for cognitive function (Fig. 2). Findings were not modified by age or sex.

### Sensitivity analysis

After excluding individuals with diabetes or past stroke, effect estimates for associations between all kidney function measures and reaction time were slightly attenuated but remained significant (Tables S9 and S10). In contrast, the associations between albuminuria and visual memory were attenuated to the null. Results were consistent after adjustment for orthogonal measures of kidney function, LDL-C and triglycerides. Regression estimates for associations in women were similar with and without restriction to postmenopausal status and after adjustment for hormone replacement therapy. Associations between eGFRcys and cognitive ability were essentially unchanged after adjustment for self-reported history of cancer, hyperthyroidism, or hypothyroidism. Parameter estimates were slightly attenuated with the additional adjustment of age^2^, but largely consistent with the primary analyses (Table S11). After adjustment for whole body fat-free mass, the magnitude of the association between eGFRcre < 60 and verbal-numeric reasoning was slightly larger, but results were otherwise similar (Table S12).

## Discussion

In this study including between 118,146 and 341,208 participants of the UKBB, markers of poor kidney function were associated with worse performance across multiple domains of cognitive function. Individuals with albuminuria scored worse on all tested measures of cognitive function including verbal-numeric reasoning, reaction time and visual memory. We observed a potential PGS by environment interaction where participants with both albuminuria and a low polygenic score for cognitive function had the lowest verbal-numeric reasoning scores. Performance on the reaction time test was worse in participants with eGFRcre < 60, as was performance on the verbal-numeric reasoning test. eGFRcys was more strongly associated with cognitive ability than eGFRcre based on serum creatinine.

Due to the unique nature of the UK Biobank cognitive tests, the clinical significance of our findings is not clear. Based on cross-sectional age coefficients, differences in reaction time with eGFRcre < 60, eGFRcys < 60 and albuminuria are comparable to an additional 1.5, 2.7, and 1.7 years of age, respectively (Figure S6a). A similar comparison would not be appropriate to interpret the verbal-numerical reasoning scores as estimates did not differ greatly as a function of cross-sectional age (a decrease of 0.01 per year of age) potentially due to age cohort effects [50]. However, differences in verbal-numeric reasoning with eGFRcre < 60, eGFRcys < 60 and albuminuria are comparable to 0.9, 1.6, and 0.8 fewer years of education, respectively (Figure S6b).

Our finding that albuminuria is associated with reduced cognitive performance is in agreement with prior studies [11, 12, 51, 52]. While the mechanism of this association is unclear, it may be related to increased vascular burden affecting both the kidney and the brain. Albuminuria is an early marker of generalized microvascular dysfunction [53] and has a linear relationship with cardiovascular disease risk [54]. In addition, albuminuria is associated with vascular dementia [55], stroke and subclinical cerebrovascular disease including white matter hyperintensities, microbleeds and enlarged perivascular spaces [55, 56]. We observed persistent significant associations between albuminuria and cognitive function after adjustment for cardiovascular disease, suggesting that pathological mechanisms may be independent of overt cardiovascular disease. While these results support the hypothesis that this association is the result of concurrent microvascular pathology in the kidney and the brain, further research is needed to clarify the relationship between the kidney damage marked by albuminuria and risk of cognitive decline.

Chronic kidney disease defined by creatinine-based eGFR has been linked with decreased cognitive ability, but the association has not been consistent [51, 57–60]. In this study, we found significant differences between eGFRcre category and cognitive performance. However, effect estimates were modest and may have limited clinical significance. The reason for the observed sex difference with regards to eGFRcre and verbal-numeric reasoning is unclear. In cross-sectional analysis, Cornelis et al. found greater age-related decreases in verbal numerical reasoning scores in men compared to women after covariate adjustment and attributed this to cohort effects [50]. This may obscure an association in older women who would be more likely to have lower eGFRcre but may have smaller age-related decreases in cognitive function compared to men in the same age group.

Cystatin C has received considerably less attention than creatinine in regard to cognitive health [14]. This study supports past research which suggests that serum cystatin C and eGFRcys may be more strongly associated with cognitive performance compared to creatinine-based measurements [20, 61]. Cystatin C-based GFR has also been shown to be a stronger predictor of cardiovascular disease outcomes [18, 62] which may mediate this association. Associations were essentially unchanged after controlling for existing cardiovascular disease in this study. This does not preclude a potential role of subclinical cardiovascular disease. On the other hand, reduced kidney function may also have direct neurodegenerative effects through inflammatory processes and accumulation of uremic toxins [46, 63]. This may be particularly relevant here as cystatin C has been related to systemic inflammation [64].

Interestingly, associations between eGFRcys category and reaction time were somewhat attenuated in older individuals. Similar age effects have been seen in observational studies examining associations between eGFRcre and mortality and ESRD [65, 66]. In older participants, the moderate-to-mild declines in kidney function observed here may have a proportionately smaller influence on cognitive function relative to other age-related comorbidities. It should also be noted that this observation may in part be due to a selection bias in which healthier older adults chose to participate in the UKBB study.

To our knowledge, there has only been one previous study that explored gene by environment interaction in the context of kidney function and cognitive performance [67]. Shin et al. found significant interaction between microalbuminuria and the *APOE* epsilon4 allele in a Korean population, where albuminuria was more strongly associated with poor cognitive performance in *APOE* epsilon4 carriers vs. noncarriers. It should be noted that SNPs contributing to the *APOE* epsilon haplotype were not used to construct PGScog, but one SNP (rs10414043) approximately 3 kb downstream of *APOE* was used in the score calculation. Taken together, the current study and that of Shin et al. suggest that a genetic susceptibility to poor cognitive performance and the presence of albuminuria may have synergistic adverse effects on brain function. Conversely, genetic factors may provide some resistance to the burden of microvascular disease on the brain. Whether the current association is mediated by gene variants that further exacerbate or attenuate the risk of microvascular dysfunction related to albuminuria is a topic for further study. Albuminuria has both genetic and environmental components [68]. The environmental component can be targeted for intervention to reduce cognitive risk. Similarly, stratification based on polygenic scores may allow clinicians to better target individuals for more aggressive treatment and intervention strategies. It is unclear why this interaction was observed for the verbal-numeric reasoning test only, but this may be a consequence of the test’s higher genetic and observational correlations with global cognitive ability relative to the reaction time and visual memory tasks [35, 69].

The observational nature of this study limited our ability to draw causal inferences. An alternative study design such as Mendelian randomization (MR), which uses genetic variants as a proxy for the exposure of interest to minimize bias due to confounding and reverse causality, may help to determine the potential causal effects of these kidney function markers. Using genetic data from the UK Biobank, we recently carried out a one-sample MR study examining the causal effects of eGFRcys, eGFRcre and ACR levels on cognitive performance [70]. Although, the results did not support causal effects of eGFRcre or eGFRcys on cognitive function outcomes, there was suggestive evidence of a causal relationship between ACR and slower reaction time and worse visual memory. Interestingly, a two-sample MR study by Chen et al. reported a significant causal association between ACR and decreased brain cortical thickness but found no such effect of eGFRcre on this outcome [71]. This lends support to a possible causal influence of albuminuria on cognitive function through alterations in brain structure. A significant limitation of these studies, however, is the difficulty in distinguishing genetic proxies that contribute to kidney function from those that are merely determinants cystatin C expression or creatinine metabolism.

There are several strengths to our study. The large size of the study population allowed us to examine gene by environment interaction which typically requires considerable sample size. We leveraged an alternate control selection approach to account for potential confounding of kidney function and cognitive performance associations through propensity-score matching without extensive loss of information due to inadequate matching which may occur in smaller samples. In addition, the extensive biochemical data allowed comparison of multiple measures of kidney function within one cohort.

Some limitations of our study should also be noted. Our analysis was restricted to participants of European ancestry which may limit generalizability to other ethnic groups. Additionally, given the voluntary nature of UKBB recruitment, the participants were generally healthier with higher socioeconomic levels than the general population [72]. It follows that the prevalence of CKD may also be comparatively lower in the UK Biobank population. However, the large overall sample size allowed for identification of an adequate number of individuals with kidney disease to characterize associations that may be applicable to broader populations. The cognitive tests in the UKBB were brief and were developed to be administered on a large scale and without supervision and may therefore not be sensitive to cognitive differences. However, the tests used here have been shown to have substantial correlation with previously validated tests in an independent sample of individuals [35]. Finally, this was a cross-sectional study limiting our ability to assess temporality. Longitudinal follow-up is required to better elucidate the temporal associations between kidney function, potential mediators such as cardiovascular disease and subsequent cognitive impairment.

## Conclusions

In summary, this study confirms prior associations between reduced kidney function and lower cognitive ability. We also show that the association between albuminuria and verbal-numeric reasoning may be modified by polygenic score for cognitive function, but results need to be replicated in independent cohorts. Low eGFR was associated with worse cognitive performance, and associations appeared stronger when GFR was estimated based on cystatin C rather than creatinine.

## Supplementary Information

**Additional file 1: Fig S1.** Histograms showing propensity score distributions between unmatched and matched samples for (a) eGFRcre<60ml/min, (b) eGFRcys<60ml/min and (c) albuminuria in individuals who completed the verbal-numeric reasoning test. Treatment refers to case status (0=control, 1=case). **Fig S2.** Histograms showing propensity score distributions between unmatched and matched samples for (a) eGFRcre<60ml/min, (b) eGFRcys<60ml/min and (c) albuminuria in individuals who completed the reaction time test. Treatment refers to case status (0=control, 1=case). **Fig S3.** Histograms showing propensity score distributions between unmatched and matched samples for (a) eGFRcre<60ml/min, (b) eGFRcys<60ml/min and (c) albuminuria in individuals who completed the visual memory test. Treatment refers to case status (0=control, 1=case). **Fig S4.** Proportional Venn diagram illustrating the degree of overlap between individuals with eGFRcys<60 eGFRcre<60 and albuminuria. Created using BioVenn web application [73]. **Fig. S5.** Predicted mean reaction time and 95% confidence intervals using eGFRcys category as a predictor grouped by age category. Abbreviations: eGFRcys, cystatin C-based estimated glomerular filtration rate. **Fig. S6.** Differences in cognitive performance on the (a) reaction time test expressed as age year equivalents and (b) the verbal-numeric reasoning test expressed as education year equivalents according to albuminuria, eGFRcys<60, and eGFRcre<60. For reaction time tests, values are based on the ratio of the coefficients for albuminuria, eGFRcys<60, or eGFRcys<60 and cross-sectional coefficients for years of age. For verbal-numeric tests, values are based on the ratio of the coefficients for albuminuria, eGFRcys<60, or eGFRcre<60 and cross-sectional coefficients for years of education. **Table S1.** Characteristics of participants with and without verbal-numeric reasoning scores. **Table S2.**Cardiovascular disease variable definitions. **Table S3.** Characteristics of study population according to albuminuria status: the UK Biobank. **Table S4.** Characteristics of study population according to eGFRcre category. **Table S5.** Characteristics of study population according to eGFRcys category. **Table S6.**Multivariable linear regression analyses of association between kidney marker exposure categories and cognitive performance. **Table S7.**Multivariable linear regression analyses of association between kidney marker exposure categories and cognitive performance adjusted for cardiovascular disease. **Table S8.**Multivariable linear regression analyses of associations between albuminuria, cognitive function polygenic score and cognitive performance. **Table S9.**Multivariable linear regression analyses of association between kidney marker exposure categories and cognitive performance excluding those with type II diabetes. **Table S10.**Multivariable linear regression analyses of association between kidney marker exposure categories and cognitive performance excluding those with past stroke. **Table S11.**Multivariable linear regression analyses of association between kidney marker exposure categories and cognitive performance adjusting for age^2^. **Table S12.**Multivariable linear regression analyses of association between kidney marker exposure categories and cognitive performance adjusting for whole-body fat free mass.

**Additional file 2: Supplementary Data 1.** Cohort descriptive statistics: The total number of participants  (% female) and mean, minimum and maximum age per cohort are indicated.  The cohort N presented here is the number of individuals that contributed to the meta-analysis.

**Additional file 3:** **Supplementary Data 2.** Single nucleotide polymorphisms used for constructing general cognitive function polygenic score with their corresponding summary statistics and imputation quality score.

## Data Availability

The UK Biobank resource is available to bona fide researchers for health-related research in the public interest. All researchers who wish to access the research resource must register with UK Biobank by completing the registration form in the Access Management System (AMS – https://bbams.ndph.ox.ac.uk/ams/).

## Abbreviations

95% CI: 95% Confidence interval
ACR: Albumin-creatinine ratio
BMI: Body Mass Index
CHD: Coronary Heart Disease
CKD: Chronic Kidney Disease
eGFR: Estimated glomerular filtration rate
eGFRcre: Creatinine-based estimated glomerular filtration rate
eGFRcys: Cystatin C-based estimated glomerular filtration rate
LDL-C: LDL-cholesterol
MS: Milliseconds
PGScog: Polygenic score for cognitive function
SNP: Single nucleotide polymorphism
T2DM: Type II diabetes mellitis
UKBB: UK Biobank
