# Supplementary material for: Markers of kidney function, genetic variation related to cognitive function, and cognitive performance in the UK Biobank
Source: BMC Nephrol. 2022 Apr 27;23:159. doi: 10.1186/s12882-022-02750-6 (PMC9047316; doi:10.1186/s12882-022-02750-6)
Supplement: Supplementary file 1 — Additional file 1: Fig S1. Histograms showing propensity score distributions between unmatched and matched samples for (a) eGFRcre<60ml/min, (b) eGFRcys<60ml/min and (c) albuminuria in individuals who completed the verbal-numeric reasoning test. Treatment refers to case status (0=control, 1=case). Fig S2. Histograms showing propensity score distributions between unmatched and matched samples for (a) eGFRcre<60ml/min, (b) eGFRcys<60ml/min and (c) albuminuria in individuals who completed the reaction time test. Treatment refers to case status (0=control, 1=case). Fig S3. Histograms showing propensity score distributions between unmatched and matched samples for (a) eGFRcre<60ml/min, (b) eGFRcys<60ml/min and (c) albuminuria in individuals who completed the visual memory test. Treatment refers to case status (0=control, 1=case). Fig S4. Proportional Venn diagram illustrating the degree of overlap between individuals with eGFRcys<60 eGFRcre<60 and albuminuria. Created using BioVenn web application [73]. Fig. S5. Predicted mean reaction time and 95% confidence intervals using eGFRcys category as a predictor grouped by age category. Abbreviations: eGFRcys, cystatin C-based estimated glomerular filtration rate. Fig. S6. Differences in cognitive performance on the (a) reaction time test expressed as age year equivalents and (b) the verbal-numeric reasoning test expressed as education year equivalents according to albuminuria, eGFRcys<60, and eGFRcre<60. For reaction time tests, values are based on the ratio of the coefficients for albuminuria, eGFRcys<60, or eGFRcys<60 and cross-sectional coefficients for years of age. For verbal-numeric tests, values are based on the ratio of the coefficients for albuminuria, eGFRcys<60, or eGFRcre<60 and cross-sectional coefficients for years of education. Table S1. Characteristics of participants with and without verbal-numeric reasoning scores. Table S2.Cardiovascular disease variable definitions. Table S3. Characteristics of study popul [file 12882_2022_2750_MOESM1_ESM.docx]

**Supplementary tables and figures:** Markers of Kidney Function, Genetic Variation Related to Cognitive Function, and Cognitive Performance in the UK Biobank

**Table S1**. Characteristics of participants with and without verbal-numeric reasoning scores

|  | **Participants with verbal-numeric reasoning scores** | **Participants without verbal-numeric reasoning scores** |
| --- | --- | --- |
| Age (years) | 57.04 (8.02) | 56.61 (7.97) |
| Male | 46.0% | 45.9% |
| Smoking status |  |  |
| Current | 9.7% | 10.6% |
| Never | 54.0% | 54.2% |
| Past | 36.3% | 35.2% |
| Some university education | 58.0% | 55.2% |
| Alcohol drinking status |  |  |
| Current | 93.6% | 93.4% |
| Never | 2.9% | 3.2% |
| Past | 3.4% | 3.4% |
| Body mass index (kg/m^2^) | 27.33 (4.76) | 27.40 (4.76) |
| LDL-c (mmol/L) | 3.57 (0.87) | 3.57 (0.87) |
| Triglycerides (mmol/L) | 1.73 (0.99) | 1.76 (1.03) |
| Hypertension | 54.9% | 55.7% |
| Type II diabetes | 5.0% | 5.0% |
| Coronary artery disease | 3.3% | 3.7% |
| History of stroke | 1.5% | 1.7% |
| Heart failure | 0.3% | 0.3% |
| Cholesterol-lowering medication | 17.6% | 16.6% |
| Antihypertensive medication | 20.3% | 20.3% |
| Reaction time (ms) | 562.94 (117.88) | 551.93 (110.80) |
| Visual memory (errors) | 1.43 (0.63) | 1.44 (0.66) |
| Albuminuria | 9.0% | 8.7% |
| eGFRcre<60ml/min | 2.3% | 2.3% |
| eGFRcys<60ml/min | 4.4% | 4.6% |

Abbreviations: eGFRcre, estimated glomerular filtration rate based on serum creatinine; eGFRcys, estimated glomerular filtration rate based on serum cystatin C; LDL-C, LDL-cholesterol

Values are shown as (%) for categorical variables and mean (SD) for continuous variables. Albuminuria was defined as a urine albumin to creatinine ratio (ACR) ≥2.5mg/mmol for men and ACR ≥3.5mg/mmol for women.

**Table S2**. Cardiovascular disease variable definitions

| **Variable** | **Definition** |
| --- | --- |
| Coronary heart disease (CHD) | Self-report of myocardial infarction (MI), coronary artery bypass grafting, coronary artery angioplasty or triple heart bypass from nurse-administered verbal interview or  Hospitalization for ICD-10 codes: (I21.0-21.4, I21.9, I22, I22.0, I22.1, I22.8, I22.9, I23, I23.0-23.6, I23.8) or ICD-9 codes: (410-412.9, 414) or  Hospitalization for OPCS-4 coded procedure: (K40-K46, K49-K50, and K75) |
| Stroke | Stroke history was centrally adjudicated by UK Biobank as self-report of stroke from nurse-administered verbal interview or hospitalization for ICD-10 codes: (I60-64) or ICD-9 codes: (430, 431, 434, 436) *(http://biobank.ctsu.ox.ac.uk/crystal/refer.cgi?id=462)* |
| Heart failure | Self-report of heart failure from nurse-administered verbal interview or  Hospitalization for ICD-10 codes: (I50, I110, I130, 132) or ICD-9 codes: (428.0, 428.9) |


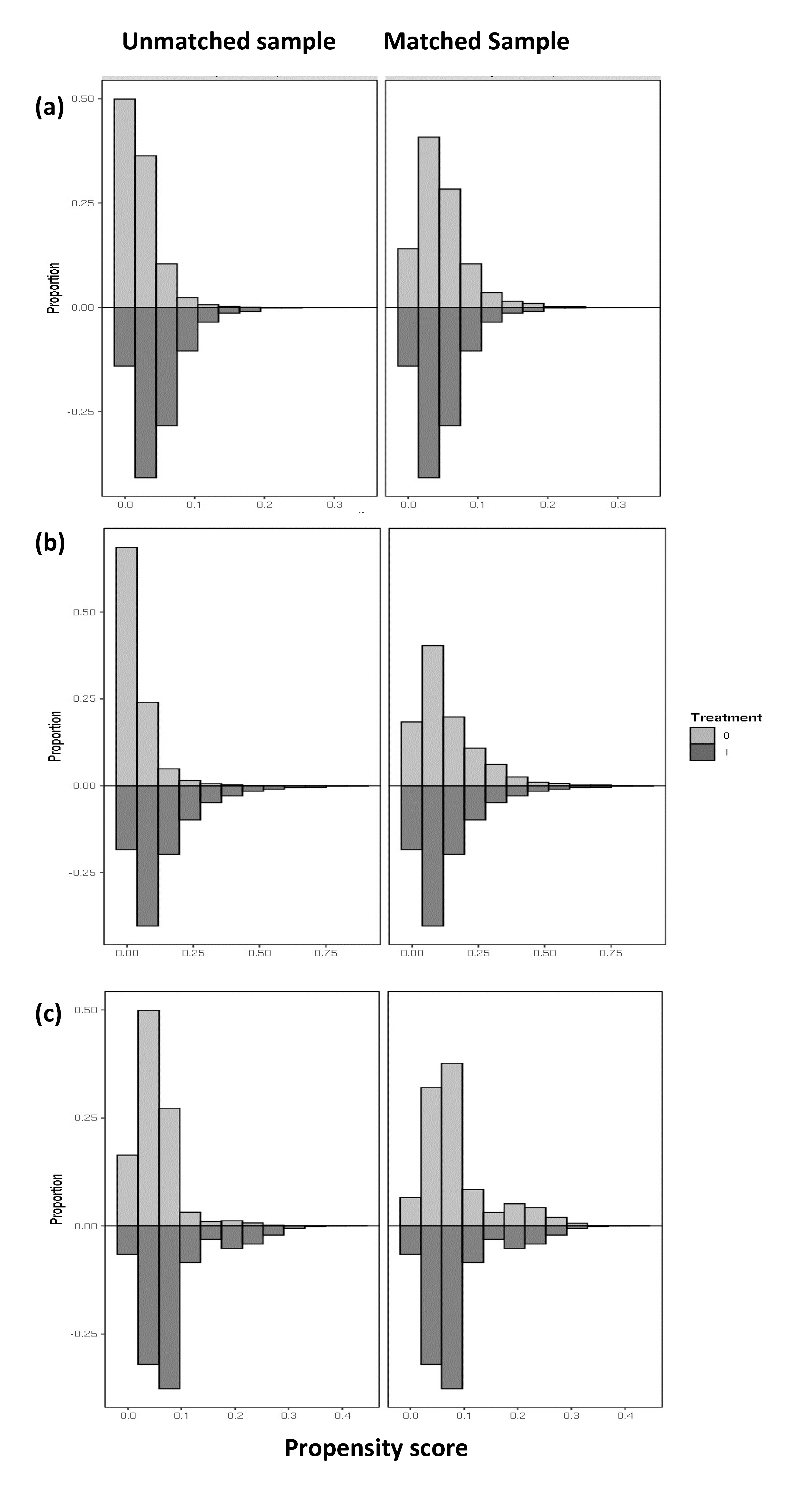


**Fig S1.** Histograms showing propensity score distributions between unmatched and matched samples for (a) eGFRcre<60ml/min, (b) eGFRcys<60ml/min and (c) albuminuria in individuals who completed the verbal-numeric reasoning test. Treatment refers to case status (0=control, 1=case).


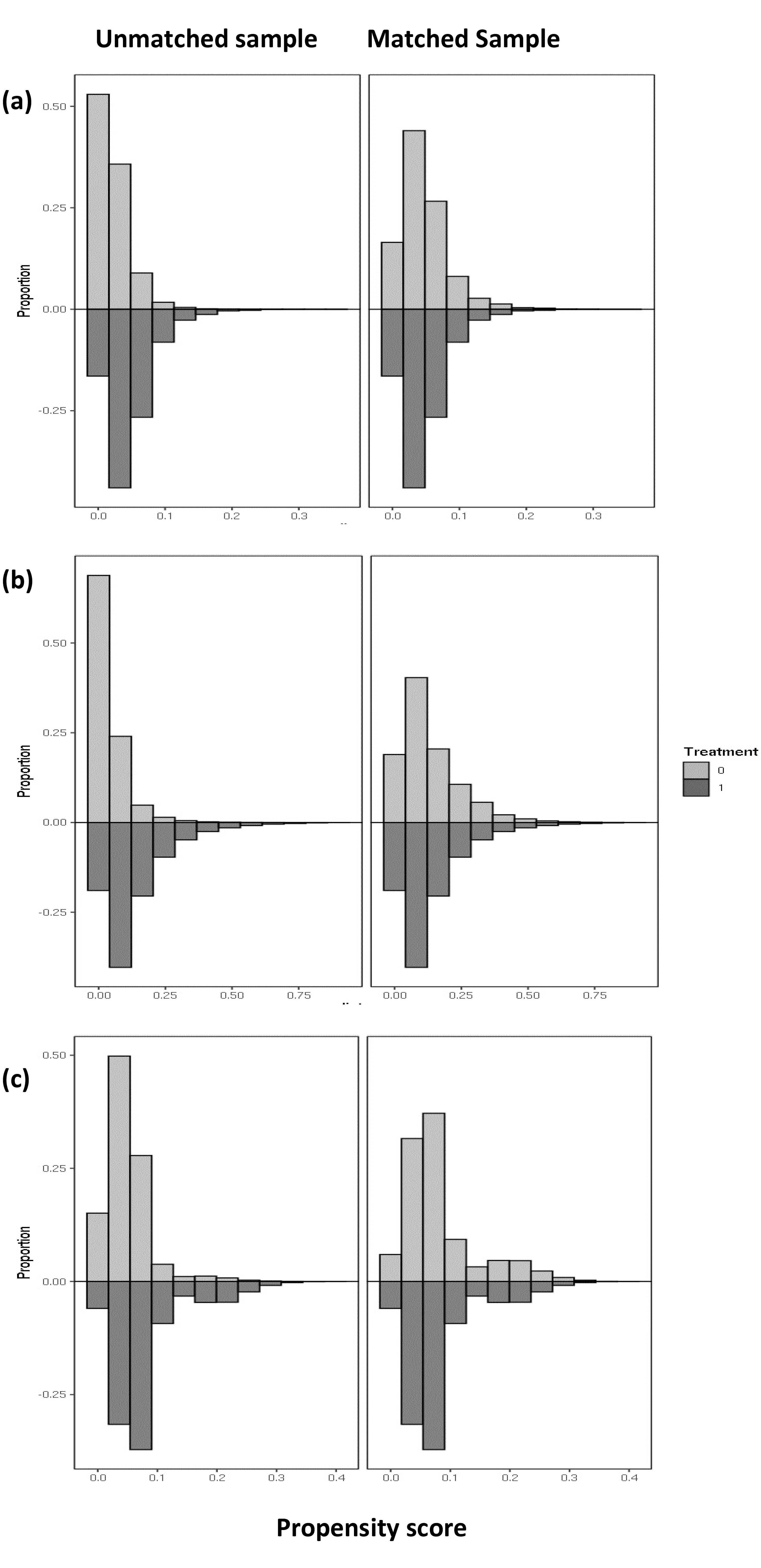


**Fig S2.** Histograms showing propensity score distributions between unmatched and matched samples for (a) eGFRcre<60ml/min, (b) eGFRcys<60ml/min and (c) albuminuria in individuals who completed the reaction time test. Treatment refers to case status (0=control, 1=case).


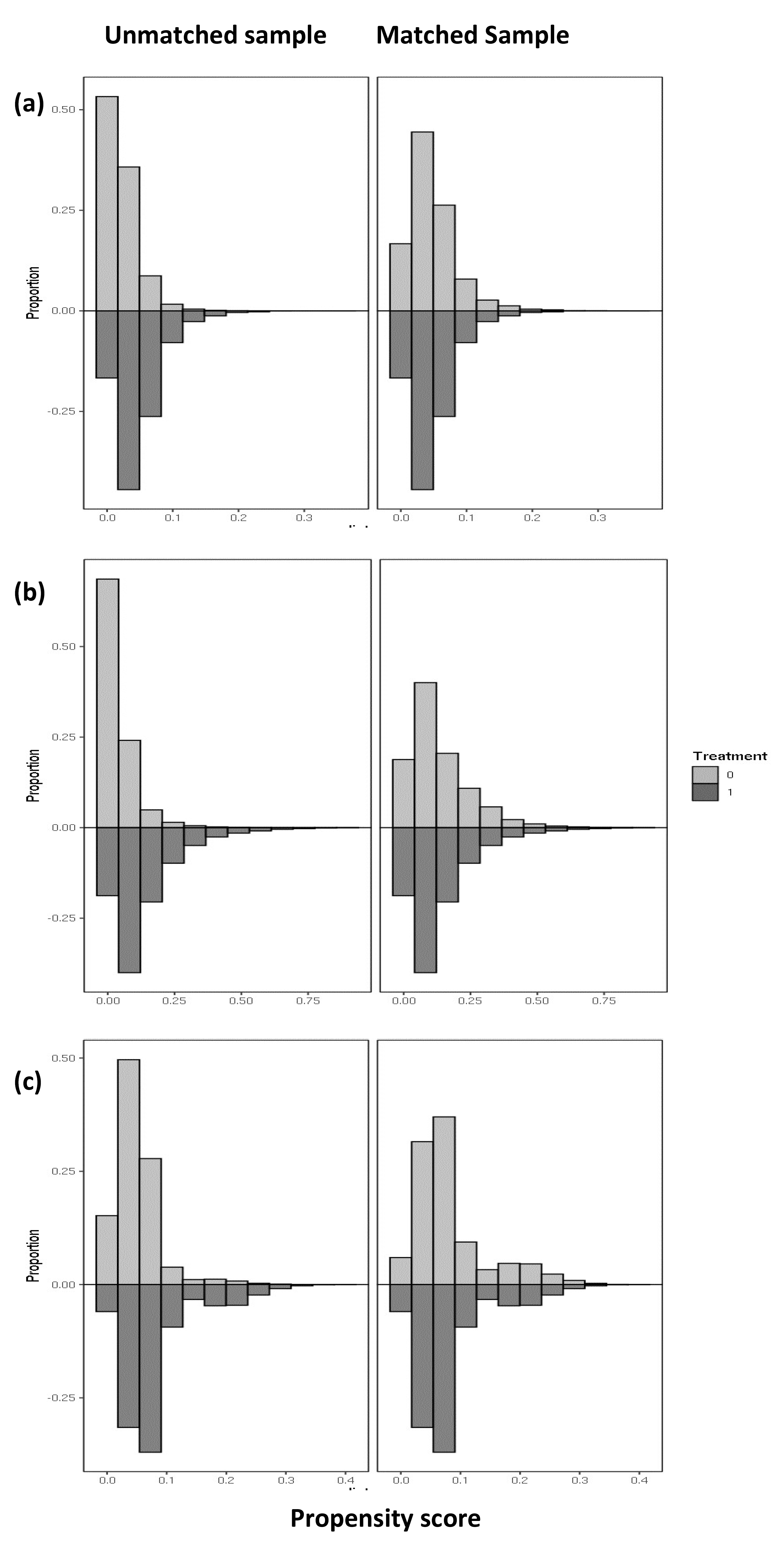


**Fig S3.** Histograms showing propensity score distributions between unmatched and matched samples for (a) eGFRcre<60ml/min, (b) eGFRcys<60ml/min and (c) albuminuria in individuals who completed the visual memory test. Treatment refers to case status (0=control, 1=case).

**Table S3**. Characteristics of study population according to albuminuria status: the UK Biobank

|  | **Albuminuria** | **No albuminuria** |
| --- | --- | --- |
|  | n=17,006 | n=324,202 |
| Age (years) | 58.98 (7.74) | 56.57 (8.00) |
| Male | 10,120 (59.5%) | 147,266 (45.4%) |
| Smoking status |  |  |
| Current | 2,360 (13.9%) | 32,522 (10.0%) |
| Never | 7,759 (45.6%) | 177,087 (54.6%) |
| Past | 6,887 (40.5%) | 114,593 (35.3%) |
| Some university education | 8,790 (51.7%) | 185,001 (57.1%) |
| Alcohol drinking status |  |  |
| Current | 15,671 (92.1%) | 303,719 (93.7%) |
| Never | 568 (3.3%) | 9,739 (3.0%) |
| Past | 767 (4.5%) | 10,744 (3.3%) |
| Body mass index (kg/m^2^) | 28.83 (5.68) | 27.26 (4.66) |
| LDL-c (mmol/L) | 3.43 (0.93) | 3.58 (0.86) |
| Triglycerides (mmol/L) | 2.03 (1.25) | 1.73 (1.00) |
| Hypertension | 13,468 (79.2%) | 174,614 (53.9%) |
| Type II diabetes | 2,835 (16.7%) | 13,761 (4.2%) |
| Coronary artery disease | 1,290 (7.6%) | 10,754 (3.3%) |
| History of stroke | 611 (3.6%) | 4,878 (1.5%) |
| Heart failure | 153 (0.9%) | 794 (0.2%) |
| Cholesterol-lowering medication | 5,630 (33.1%) | 51,500 (15.9%) |
| Antihypertensive medication | 7,132 (41.9%) | 61,483 (19.0%) |

Abbreviations: eGFR, estimated glomerular filtration rate; LDL-C, LDL-cholesterol

Values are shown as n (%) for categorical variables and mean (SD) for continuous variables. Albuminuria was defined as a urine albumin to creatinine ratio (ACR) ≥2.5mg/mmol for men and ACR ≥3.5mg/mmol for women.

All characteristics are significantly different by albuminuria status.

| **Table S4.** Characteristics of study population according to eGFRcre category | | |
| --- | --- | --- |
|  | **eGFRcre <60 ml/min** | **eGFRcre ≥60 ml/min** |
|  | n=7,605 | n=333,603 |
| Age (years) | 62.79 (5.76) | 56.55 (8.00) |
| Male | 3,534 (46.5%) | 153,852 (46.1%) |
| Smoking status |  |  |
| Current | 599 (7.9%) | 34,283 (10.3%) |
| Never | 3,665 (48.2%) | 181,181 (54.3%) |
| Past | 3,341 (43.9%) | 118,139 (35.4%) |
| Some university education | 3,558 (46.8%) | 190,233 (57.0%) |
| Alcohol drinking status |  |  |
| Current | 6,783 (89.2%) | 312,607 (93.7%) |
| Never | 419 (5.5%) | 9,888 (3.0%) |
| Past | 403 (5.3%) | 11,108 (3.3%) |
| Body mass index (kg/m^2^) | 29.10 (5.23) | 27.30 (4.71) |
| LDL-c (mmol/L) | 3.32 (0.95) | 3.58 (0.86) |
| Triglycerides (mmol/L) | 2.04 (1.09) | 1.74 (1.02) |
| Hypertension | 5,895 (77.5%) | 182,187 (54.6%) |
| Type II diabetes | 1,002 (13.2%) | 15,594 (4.7%) |
| Coronary artery disease | 912 (12.0%) | 11,132 (3.3%) |
| History of stroke | 397 (5.2%) | 5,092 (1.5%) |
| Heart failure | 159 (2.1%) | 788 (0.2%) |
| Cholesterol-lowering medication | 3,249 (42.7%) | 53,881 (16.2%) |
| Antihypertensive medication | 4,080 (53.7%) | 64,535 (19.3%) |

Abbreviations: eGFR, estimated glomerular filtration rate; LDL-C, LDL-cholesterol

Values are shown as n (%) for categorical variables and mean (SD) for continuous variables.

All characteristics are significantly different by eGFRcre category except sex (p-value=0.55).

| **Table S5.** Characteristics of study population according to eGFRcys category | | |
| --- | --- | --- |
|  | **eGFRcys<60 ml/min** | **eGFRcys ≥60 ml/min** |
|  | n=14,986 | n=326,222 |
| Age (years) | 63.21 (5.28) | 56.39 (7.98) |
| Male | 7,104 (47.4%) | 150,282 (46.1%) |
| Smoking status |  |  |
| Current | 2,466 (16.5%) | 32,416 (9.9%) |
| Never | 6,356 (42.4%) | 178,490 (54.7%) |
| Past | 6,164 (41.1%) | 115,316 (35.3%) |
| Some university education | 6,163 (41.1%) | 187,628 (57.5%) |
| Alcohol drinking status |  |  |
| Current | 12,912 (86.2%) | 306,478 (93.9%) |
| Never | 1,011 (6.7%) | 9,296 (2.8%) |
| Past | 1,063 (7.1%) | 10,448 (3.2%) |
| Body mass index (kg/m^2^) | 30.62 (5.97) | 27.19 (4.61) |
| LDL-c (mmol/L) | 3.37 (0.96) | 3.58 (0.86) |
| Triglycerides (mmol/L) | 2.12 (1.11) | 1.73 (1.01) |
| Hypertension | 12,012 (80.2%) | 176,070 (54.0%) |
| Type II diabetes | 2,224 (14.8%) | 14,372 (4.4%) |
| Coronary artery disease | 1,735 (11.6%) | 10,309 (3.2%) |
| History of stroke | 789 (5.3%) | 4,700 (1.4%) |
| Heart failure | 269 (1.8%) | 678 (0.2%) |
| Cholesterol-lowering medication | 5,955 (39.7%) | 51,175 (15.7%) |
| Antihypertensive medication | 8,105 (54.1%) | 60,510 (18.5%) |

Abbreviations: eGFR, estimated glomerular filtration rate; LDL-C, LDL-cholesterol

Values are shown as n (%) for categorical variables and mean (SD) for continuous variables.

All characteristics are significantly different by eGFRcys category.

**Fig S4.** Proportional Venn diagram illustrating the degree of overlap between individuals with eGFRcys<60 eGFRcre<60 and albuminuria. Created using BioVenn web application (73).

**Table S6**. Multivariable linear regression analyses of association between kidney marker exposure categories and cognitive performance

|  | **Verbal-numeric reasoning** | | **Reaction time** | | **Visual memory** | |
| --- | --- | --- | --- | --- | --- | --- |
|  | $\boldsymbol{\beta}$  **(95%CI)** | **p-value** | $\boldsymbol{\beta}$  **(95%CI)** | **p-value** | $\boldsymbol{\beta}$  **(95%CI)** | **p-value** |
| **Albuminuria** |  |  |  |  |  |  |
| All | -0.09 (-0.14 to -0.04) | <0.001^b^ | 7.06 (5.42 to 8.69) | <0.001^b^ | 0.013 (0.003 to 0.023) | 0.01 |
| Women | -0.08 (-0.15 to -0.01) | 0.03 | 5.32 (2.79 to 7.85) | <0.001^b^ | 0.014 (-0.001 to 0.029) | 0.06 |
| Men | -0.08 (-0.15 to -0.02) | 0.02 | 8.75 (6.62 to 10.89) | <0.001^b^ | 0.012 (-0.001 to 0.025) | 0.07 |
| p-interaction^a^ |  | 0.52 |  | 0.29 |  | 0.89 |
|  |  |  |  |  |  |  |
| **eGFRcr < 60ml/min** |  |  |  |  |  |  |
| All | -0.11 (-0.18 to -0.03) | <0.001^b^ | 6.08 (3.66 to 8.49) | <0.001^b^ | -0.005 (-0.02 to 0.009) | 0.47 |
| Women | -0.05 (-0.15 to 0.05) | 0.32 | 4.67 (1.36 to 7.98) | 0.005^b^ | -0.011 (-0.031 to 0.008) | 0.25 |
| Men | -0.18 (-0.29 to -0.07) | 0.002^b^ | 7.79 (4.26 to 11.33) | <0.001^b^ | 0.002 (-0.019 to 0.023) | 0.86 |
| p-interaction^a^ |  | 0.01 |  | 0.69 |  | 0.20 |
|  |  |  |  |  |  |  |
| **eGFRcys < 60ml/min** |  |  |  |  |  |  |
| All | -0.21 (-0.27 to -0.16) | <0.001^b^ | 11.21 (9.44 to 12.99) | <0.001^b^ | -0.002 (-0.013 to 0.008) | 0.71 |
| Women | -0.18 (-0.25 to -0.11) | <0.001^b^ | 11.29 (8.84 to 13.75) | <0.001^b^ | -0.001 (-0.015 to 0.014) | 0.92 |
| Men | -0.25 (-0.33 to -0.16) | <0.001^b^ | 11.15 (8.58 to 13.72) | <0.001^b^ | -0.004 (-0.019 to 0.012) | 0.65 |
| p-interaction^a^ |  | 0.09 |  | 0.31 |  | 0.82 |

Models adjusted for age, sex, education, Townsend deprivation index, country of birth, physical activity, hypertension, diabetes status, alcohol use, smoking status, body mass index, lipid lowering drugs, and antihypertensive drugs.

Abbreviations: eGFRcre, creatinine-based estimated glomerular filtration rate; eGFRcys, cystatin C based estimated glomerular filtration rate.

^a^p-value for marker by sex interaction.

^b^p-value significant after Bonferroni correction.

**Table S7.** Multivariable linear regression analyses of association between kidney marker exposure categories and cognitive performance adjusted for cardiovascular disease

|  | **Verbal-numeric reasoning** | | **Reaction time** | | **Visual memory** | |
| --- | --- | --- | --- | --- | --- | --- |
|  | $\boldsymbol{\beta}$  **(95%CI)** | **p-value** | $\boldsymbol{\beta}$  **(95%CI)** | **p-value** | $\boldsymbol{\beta}$  **(95%CI)** | **p-value** |
| **Albuminuria** |  |  |  |  |  |  |
| All | -0.09 (-0.14 to -0.04) | <0.001^b^ | 5.54 (3.03 to 8.05) | <0.001^b^ | 0.013 (0.004 to 0.023) | 0.01 |
| Women | -0.08(-0.15 to 0.004) | 0.04 | 4.99 (2.46 to 7.52) | <0.001^b^ | 0.014 (-0.001 to 0.029) | 0.06 |
| Men | -0.08 (-0.15 to -0.01) | 0.02 | 8.27 (6.14 to 10.40) | <0.001^b^ | 0.012 (-0.001 to 0.025) | 0.07 |
| p-interaction^a^ |  | 0.61 |  | 0.27 |  | 0.90 |
|  |  |  |  |  |  |  |
| **eGFRcr < 60ml/min** |  |  |  |  |  |  |
| All | -0.09 (-0.16 to -0.01) | 0.02 | 5.03 (1.75 to 8.31) | 0.003^b^ | -0.005 (-0.020 to 0.009) | 0.47 |
| Women | -0.04 (-0.13 to 0.06) | 0.50 | 3.73 (0.42 to 7.04) | 0.03 | -0.012 (-0.031 to 0.007) | 0.22 |
| Men | -0.15 (-0.26 to -0.04) | 0.008 | 5.83 (2.29 to 9.37) | 0.001^b^ | 0.002 (-0.019 to 0.024) | 0.83 |
| p-interaction^a^ |  | 0.03 |  | 0.08 |  | 0.82 |
|  |  |  |  |  |  |  |
| **eGFRcys < 60ml/min** |  |  |  |  |  |  |
| All | -0.20 (-0.25 to -0.14) | <0.001^b^ | 11.52 (9.1 to 13.93) | <0.001^b^ | -0.002 (-0.013 to 0.008) | 0.69 |
| Women | -0.17 (-0.25 to -0.1) | <0.001^b^ | 10.46 (8.0 to 12.92) | <0.001^b^ | -0.001 (-0.016 to 0.013) | 0.85 |
| Men | -0.23 (-0.31 to -0.15) | <0.001^b^ | 9.50 (6.92 to 12.08) | <0.001^b^ | -0.003 (-0.019 to 0.012) | 0.68 |
| p-interaction^a^ |  | 0.08 |  | 0.81 |  | 0.20 |

Models adjusted for age, sex, education, Townsend deprivation index, country of birth, physical activity, hypertension, diabetes status, alcohol use, smoking status, body mass index, lipid lowering drugs, antihypertensive drugs, coronary artery disease, stroke and heart failure.

Abbreviations: eGFRcre, creatinine-based estimated glomerular filtration rate; eGFRcys, cystatin C based estimated glomerular filtration rate.

^a^p-value for marker by sex interaction.

^b^p-value significant after Bonferroni correction.

**Table S8.** Multivariable linear regression analyses of associations between albuminuria, cognitive function polygenic score and cognitive performance

|  | **Verbal-numeric memory** | | **Reaction time** | | **Visual memory** | |
| --- | --- | --- | --- | --- | --- | --- |
|  | $\boldsymbol{\beta}$ **(95%CI)** | **p-value** | $\boldsymbol{\beta}$ **(95%CI)** | **p-value** | $\boldsymbol{\beta}$ **(95%CI)** | **p-value** |
| **Model 1** |  |  |  |  |  |  |
| Albuminuria | -0.21(-0.26,-0.16) | <0.0001 | 9.93(8.31,11.55) | <0.0001 | 0.009(-0.001,0.018) | 0.07 |
| PRScog | 0.11(0.10-0.12) | <0.0001 | -0.85(-1.21,-0.50) | <0.0001 | -0.007(-0.01,-0.005) | <0.0001 |
| **Model 2** |  |  |  |  |  |  |
| Albuminuria | -0.21(-0.26,-0.16) | <0.0001 | 9.93(8.31,11.55) | <0.0001 | 0.009(-0.001,0.018) | 0.07 |
| PRScog | 0.11(0.10,0.12) | <0.0001 | -0.87(-1.23,-0.50) | <0.0001 | -0.008(-0.01,-0.005) | <0.0001 |
| Albuminuria x PRScog | 0.06(0.005,0.11) | 0.03 | 0.22(-1.38,1.82) | 0.79 | 0.002(-0.007,0.012) | 0.62 |
| **Model 3** |  |  |  |  |  |  |
| Albuminuria | -0.12(-0.17,-0.07) | <0.0001 | 8.08(6.47,9.70) | <0.0001 | 0.006(-0.004,0.015) | 0.24 |
| PRScog | 0.08(0.07,0.09) | <0.0001 | -0.58(-0.94,-0.22) | 0.002 | -0.007(-0.009,-0.005) | <0.0001 |
| Albuminuria x PRScog | 0.06(0.01,0.11) | 0.01 | 0.28(-1.32,1.87) | 0.74 | 0.002(-0.007,0.012) | 0.61 |
| **Model 4** |  |  |  |  |  |  |
| Albuminuria | -0.09(-0.14,-0.04) | 0.0009 | 7.00(5.38,8.63) | <0.0001 | 0.013(0.003,0.022) | 0.01 |
| PRScog | 0.08(0.07,0.09) | <0.0001 | -0.57(-0.93,-0.21) | 0.002 | -0.007(-0.009,-0.005) | <0.0001 |
| Albuminuria x PRScog | 0.06(0.02,0.11) | 0.009 | 0.28(-1.31,1.87) | 0.73 | 0.003(-0.006,0.012) | 0.54 |
| **Model 5** |  |  |  |  |  |  |
| Albuminuria | -0.08(-0.14,-0.04) | 0.002 | 6.91(5.28,8.53) | <0.0001 | 0.013(0.004,0.024) | 0.008 |
| PRScog | 0.08(0.07,0.09) | <0.0001 | -0.57(-0.93,-0.21) | 0.002 | -0.007(-0.009,-0.005) | <0.0001 |
| Albuminuria x PRScog | 0.07(0.02,0.12) | 0.009 | 0.37(-1.22,1.96) | 0.65 | 0.002(-0.007,0.011) | 0.68 |

Model 1: main effects of albuminuria and PRScog adjusted for age, sex, country of birth, and principal components

Model 2: model 1+ the albuminuria by PRScog interaction term

Model 3: model 2 + education and Townsend deprivation score

Model 4: model 3 + physical activity, hypertension, diabetes status, alcohol use, smoking status, body mass index, lipid lowering drugs, and antihypertensive drugs.

Model 5: model 4 + age^2^

Abbreviations: eGFRcre, creatinine-based estimated glomerular filtration rate; eGFRcys, cystatin C based estimated glomerular filtration rate.

**Table S9.** Multivariable linear regression analyses of association between kidney marker exposure categories and cognitive performance excluding those with type II diabetes

|  | **Verbal-numeric reasoning** | | **Reaction time** | | **Visual memory** | |
| --- | --- | --- | --- | --- | --- | --- |
|  | $\boldsymbol{\beta}$  **(95%CI)** | **p-value** | $\boldsymbol{\beta}$  **(95%CI)** | **p-value** | $\boldsymbol{\beta}$  **(95%CI)** | **p-value** |
| **Albuminuria** |  |  |  |  |  |  |
| All | -0.11 (-0.16 to -0.05) | <0.001^b^ | 5.89 (4.13 to 7.65) | <0.001^b^ | 0.010 (-0.001 to 0.02) | 0.07 |
| Women | -0.10 (-0.17 to -0.02) | 0.01 | 4.15 (1.51 to 6.79) | <0.001^b^ | 0.011 (-0.005 to 0.026) | 0.17 |
| Men | -0.10 (-0.17 to -0.02) | 0.01 | 7.76 (5.4 to 10.11) | <0.001^b^ | 0.008 (-0.007 to 0.022) | 0.29 |
| p-interaction^a^ |  | 0.64 |  | 0.13 |  | 0.88 |
|  |  |  |  |  |  |  |
| **eGFRcr < 60ml/min** |  |  |  |  |  |  |
| All | -0.11 (-0.19 to -0.03) | 0.007^b^ | 5.54 (2.96 to 8.11) | <0.001^b^ | -0.006 (-0.021 to 0.01) | 0.46 |
| Women | -0.05 (-0.16 to 0.05) | 0.30 | 4.07 (0.61 to 7.53) | 0.02 | -0.015 (-0.035 to 0.005) | 0.14 |
| Men | -0.18 (-0.30 to -0.06) | 0.003^b^ | 7.53 (3.68 to 11.39) | 0.001^b^ | 0.006 (-0.017 to 0.03) | 0.61 |
| p-interaction^a^ |  | 0.02 |  | 0.80 |  | 0.09 |
|  |  |  |  |  |  |  |
| **eGFRcys < 60ml/min** |  |  |  |  |  |  |
| All | -0.21 (-0.27 to -0.15) | <0.001^b^ | 10.45 (8.54 to 12.35) | <0.001^b^ | -0.003 (-0.014 to 0.009) | 0.65 |
| Women | -0.19 (-0.27 to -0.11) | <0.001^b^ | 10.80 (8.21 to 13.40) | <0.001^b^ | -0.003 (-0.018 to 0.012) | 0.74 |
| Men | -0.23 (-0.32 to -0.14) | <0.001^b^ | 10.08 (7.27 to 12.88) | <0.001^b^ | -0.003 (-0.02 to 0.014) | 0.76 |
| p-interaction^a^ |  | 0.07 |  | 0.07 |  | 0.60 |

Models adjusted for age, sex, education, Townsend deprivation index, country of birth, physical activity, hypertension, alcohol use, smoking status, body mass index, lipid lowering drugs, and antihypertensive drugs.

Abbreviations: eGFRcre, creatinine-based estimated glomerular filtration rate; eGFRcys, cystatin C based estimated glomerular filtration rate.

^a^p-value for marker by sex interaction.

^b^p-value significant after Bonferroni correction.

**Table S10.** Multivariable linear regression analyses of association between kidney marker exposure categories and cognitive performance excluding those with past stroke

|  | **Verbal-numeric reasoning** | | **Reaction time** | | **Visual memory** | |
| --- | --- | --- | --- | --- | --- | --- |
|  | $\boldsymbol{\beta}$ **(95%CI)** | **p-value** | $\boldsymbol{\beta}$ **(95%CI)** | **p-value** | $\boldsymbol{\beta}$ **(95%CI)** | **p-value** |
| **Albuminuria** |  |  |  |  |  |  |
| All | -0.08 (-0.13 to -0.03) | 0.003^b^ | 6.41 (4.77 to 8.06) | <0.001^b^ | 0.008 (-0.002 to 0.019) | 0.13 |
| Women | -0.08(-0.15 to -0.001) | 0.05 | 4.42(1.88 to 6.97) | 0.01 | 0.009 (-0.006 to 0.025) | 0.23 |
| Men | -0.08 (-0.14 to -0.01) | 0.03 | 8.35 (6.20 to 10.51) | <0.001^b^ | 0.006 (-0.008 to 0.021) | 0.39 |
| p-interaction^a^ |  | 0.56 |  | 0.12 |  | 0.86 |
|  |  |  |  |  |  |  |
| **eGFRcr < 60ml/min** |  |  |  |  | -0.002 (-0.023 to 0.019) |  |
| All | -0.10 (-0.18 to -0.02) | 0.01 | 4.91 (2.45 to 7.37) | <0.001^b^ | -0.006 (-0.022 to 0.01) | 0.46 |
| Women | -0.04 (-0.14 to 0.06) | 0.40 | 4.12 (0.78 to 7.46) | 0.04 | -0.013 (-0.034 to 0.007) | 0.19 |
| Men | -0.17 (-0.29 to -0.05) | 0.004^b^ | 5.95 (2.31 to 9.58) | 0.004^b^ | 0.004 (-0.02 to 0.029) | 0.73 |
| p-interaction^a^ |  | 0.01 |  | 0.85 |  | 0.14 |
|  |  |  |  |  |  |  |
| **eGFRcys < 60ml/min** |  |  |  |  |  |  |
| All | -0.20 (-0.25 to -0.14) | <0.001^b^ | 9.98 (8.17 to 11.79) | <0.001^b^ | -0.004 (-0.015 to 0.008) | 0.51 |
| Women | -0.18 (-0.26 to -0.1) | <0.001^b^ | 10.60 (8.11 to 13.08) | <0.001^b^ | -0.004 (-0.019 to 0.012) | 0.62 |
| Men | -0.22 (-0.3 to -0.13) | <0.001^b^ | 9.27 (6.64 to 11.91) | <0.001^b^ | -0.004 (-0.021 to 0.014) | 0.66 |
| p-interaction^a^ |  | 0.08 |  | 0.08 |  | 0.60 |

Models adjusted for age, sex, education, Townsend deprivation index, country of birth, physical activity, hypertension, diabetes status, alcohol use, smoking status, body mass index, lipid lowering drugs, and antihypertensive drugs.

Abbreviations: eGFRcre, creatinine-based estimated glomerular filtration rate; eGFRcys, cystatin C based estimated glomerular filtration rate.

^a^p-value for marker by sex interaction.

^b^p-value significant after Bonferroni correction.

**Table S11**. Multivariable linear regression analyses of association between kidney marker exposure categories and cognitive performance adjusting for age^2^.

|  | **Verbal-numeric reasoning** | | **Reaction time** | | **Visual memory** | |
| --- | --- | --- | --- | --- | --- | --- |
|  | $\boldsymbol{\beta}$  **(95%CI)** | **p-value** | $\boldsymbol{\beta}$  **(95%CI)** | **p-value** | $\boldsymbol{\beta}$  **(95%CI)** | **p-value** |
| **Albuminuria** |  |  |  |  |  |  |
| All | -0.08 (-0.13 to -0.03) | <0.001^b^ | 6.97 (5.34 to 8.60) | <0.001^b^ | 0.013 (0.004 to 0.023) | 0.01 |
| Women | -0.08 (-0.15 to -0.01) | 0.03 | 5.21 (2.68 to 7.74) | <0.001^b^ | 0.014 (-0.001 to 0.029) | 0.06 |
| Men | -0.07 (-0.14 to -0.01) | 0.02 | 8.63 (6.49 to 10.76) | <0.001^b^ | 0.011 (-0.002 to 0.024) | 0.10 |
| p-interaction^a^ |  | 0.58 |  | 0.18 |  | 0.51 |
|  |  |  |  |  |  |  |
| **eGFRcr < 60ml/min** |  |  |  |  |  |  |
| All | -0.10 (-0.17 to -0.02) | 0.01 | 5.70 (3.26 to 8.10) | <0.001^b^ | -0.005 (-0.019 to 0.009) | 0.50 |
| Women | -0.04 (-0.13 to 0.06) | 0.46 | 4.30 (0.99 to 7.61) | 0.01 | -0.011 (-0.030 to 0.008) | 0.27 |
| Men | -0.17 (-0.28 to -0.06) | 0.003^b^ | 7.35 (3.80 to 10.89) | <0.001^b^ | 0.002 (-0.019 to 0.024) | 0.84 |
| p-interaction^a^ |  | 0.01 |  | 0.72 |  | 0.21 |
|  |  |  |  |  |  |  |
| **eGFRcys < 60ml/min** |  |  |  |  |  |  |
| All | -0.20 (-0.25 to -0.14) | <0.001^b^ | 10.82 (9.04 to 12.61) | <0.001^b^ | -0.002 (-0.012 to 0.009) | 0.71 |
| Women | -0.17 (-0.24 to -0.09) | <0.001^b^ | 10.91 (8.44 to 13.38) | <0.001^b^ | -0.001 (-0.015 to 0.014) | 0.97 |
| Men | -0.23 (-0.31 to -0.15) | <0.001^b^ | 10.74 (8.15 to 13.31) | <0.001^b^ | -0.003 (-0.019 to 0.012) | 0.67 |
| p-interaction^a^ |  | 0.01 |  | 0.30 |  | 0.87 |

Models adjusted for age, age^2^, sex, education, Townsend deprivation index, country of birth, physical activity, hypertension, diabetes status, alcohol use, smoking status, body mass index, lipid lowering drugs, and antihypertensive drugs.

Abbreviations: eGFRcre, creatinine-based estimated glomerular filtration rate; eGFRcys, cystatin C based estimated glomerular filtration rate.

^a^p-value for marker by sex interaction.

^b^p-value significant after Bonferroni correction.

**Table S12**. Multivariable linear regression analyses of association between kidney marker exposure categories and cognitive performance adjusting for whole-body fat free mass

|  | **Verbal-numeric reasoning** | | **Reaction time** | | **Visual memory** | |
| --- | --- | --- | --- | --- | --- | --- |
|  | $\boldsymbol{\beta}$  **(95%CI)** | **p-value** | $\boldsymbol{\beta}$  **(95%CI)** | **p-value** | $\boldsymbol{\beta}$  **(95%CI)** | **p-value** |
| **Albuminuria** |  |  |  |  |  |  |
| All | -0.09 (-0.14 to -0.04) | <0.001^b^ | 7.01 (5.40 to 8.66) | <0.001 ^b^ | 0.013 (0.003 to 0.023) | 0.008 ^b^ |
| Women | -0.08 (-0.15 to -0.004) | 0.04 | 5.21 (2.69 to 7.74) | <0.001 ^b^ | 0.014 (-0.001 to 0.029) | 0.06 |
| Men | -0.08 (-0.15 to -0.01) | 0.03 | 8.67 (6.53 to 10.80) | <0.001 ^b^ | 0.012 (-0.001 to 0.025) | 0.07 |
| p-interaction^a^ |  | 0.46 |  | 0.17 |  | 0.90 |
|  |  |  |  |  |  |  |
| **eGFRcr < 60ml/min** |  |  |  |  |  |  |
| All | -0.13 (-0.21 to -0.06) | <0.001 ^b^ | 5.77 (3.36 to 8.20) | <0.001 ^b^ | -0.006 (-0.02 to 0.009) | 0.43 |
| Women | -0.09 (-0.19 to 0.004) | 0.07 | 4.38 (1.07 to 7.70) | 0.01 | -0.011 (-0.031 to 0.008) | 0.25 |
| Men | -0.19 (-0.30 to -0.08) | 0.001 ^b^ | 8.15 (4.57 to 11.72) | <0.001 ^b^ | 0.001 (-0.021 to 0.01) | 0.98 |
| p-interaction^a^ |  | 0.02 |  | 0.28 |  | 0.20 |
|  |  |  |  |  |  |  |
| **eGFRcys < 60ml/min** |  |  |  |  |  |  |
| All | -0.21 (-0.27 to -0.16) | <0.001 ^b^ | 11.01 (9.23 to 12.80) | <0.001 ^b^ | -0.003 (-0.011 to 0.013) | 0.91 |
| Women | -0.19 (-0.27 to -0.12) | <0.001 ^b^ | 11.08 (8.60 to 13.56) | <0.001 ^b^ | -0.001 (-0.015 to 0.013) | 0.92 |
| Men | -0.24 (-0.32 to -0.15) | <0.001 ^b^ | 10.94 (8.15 to 13.30) | <0.001 ^b^ | -0.004 (-0.019 to 0.012) | 0.53 |
| p-interaction^a^ |  | 0.03 |  | 0.46 |  | 0.92 |

Models adjusted for age, sex, education, Townsend deprivation index, country of birth, physical activity, hypertension, diabetes status, alcohol use, smoking status, body mass index, lipid lowering drugs, antihypertensive drugs, and whole-body fat free mass.

Abbreviations: eGFRcre, creatinine-based estimated glomerular filtration rate; eGFRcys, cystatin C based estimated glomerular filtration rate.

^a^p-value for marker by sex interaction.

^b^p-value significant after Bonferroni correction.


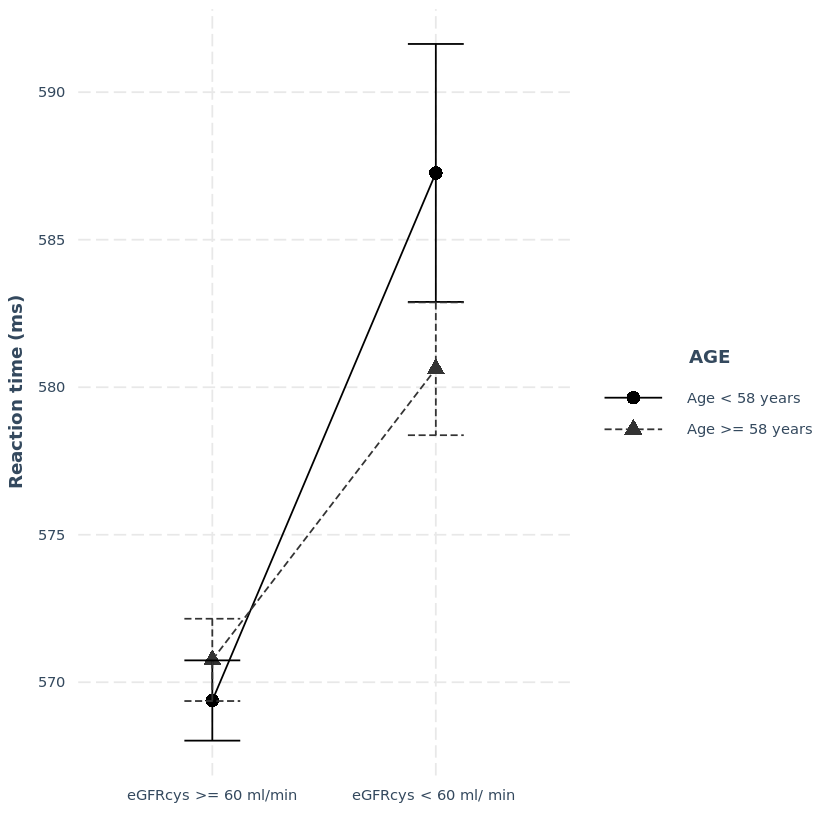


**Fig. S5**. Predicted mean reaction time and 95% confidence intervals using eGFRcys category as a predictor

grouped by age category. Abbreviations: eGFRcys, cystatin C based estimated glomerular filtration rate

**Fig. S6**. Differences in cognitive performance on the (a) reaction time test expressed as age year equivalents and (b) the verbal-numeric reasoning test expressed as education year equivalents according to albuminuria, eGFRcys<60, and eGFRcre<60. For reaction time tests, values are based on the ratio of the coefficients for albuminuria, eGFRcys<60, or eGFRcys<60 and cross-sectional coefficients for years of age. For verbal-numeric tests, values are based on the ratio of the coefficients for albuminuria, eGFRcys<60, or eGFRcre<60 and cross-sectional coefficients for years of education.
